# The effects of testicular aging on Leydig cells and the application of stem cells in restoring Leydig cells function

**DOI:** 10.1186/s12958-025-01487-9

**Published:** 2025-11-19

**Authors:** Wen-bo Zhu, Han-bin Zhao, Guang-zhao Wang, Zu-long Wang, Shi-qi Wang

**Affiliations:** 1 The First Clinical Medical College, Henan University of Chinese Medicine, Henan, 450046 China; 2 Department of Andrology, The First Affiliated Hospital of Henan University of Chinese Medicine, Henan, 450046 China; 3 Department of Cancer Diagnosis and Treatment Center, The First Affiliated Hospital of Henan University of Chinese Medicine, Henan, 450046 China

**Keywords:** Testicular aging, Leydig cells, Stem leydig cells, Mesenchymal stem cells, Induced pluripotent stem cells

## Abstract

With the global population aging at an accelerated pace, testicular aging and its associated male health issues have garnered increasing attention. Testicular aging encompasses both age-related physiological decline and premature or pathological aging caused by various pathogenic factors. It is primarily characterized by reduced androgen secretion (mainly testosterone) and impaired spermatogenesis. Leydig cells, the key cells in the testes responsible for synthesizing and secreting testosterone, play a critical role in maintaining testicular function, and their dysfunction is one of the major contributors to testicular functional decline. In recent years, stem cell technology has demonstrated significant potential in restoring Leydig cell function. Stem cells possess the ability to differentiate into Leydig-like cells, which can restore testosterone secretion, improve the testicular microenvironment, and promote the regeneration of endogenous Leydig cells. Currently, various types of stem cells, such as stem leydig cells, mesenchymal stem cells, and induced pluripotent stem cells, have been successfully directed to differentiate into Leydig-like cells. This article provides an in-depth analysis of the mechanisms by which testicular aging affects Leydig cells, with a particular focus on the application of stem cell technology in restoring Leydig cell function. Additionally, it explores potential strategies to address these issues, aiming to offer novel perspectives and approaches for the prevention and treatment of testicular aging.

## Introduction

With the intensifying global issue of population aging, China, as the most populous country in the world, is facing a significant aging crisis accompanied by a rising prevalence of age-related diseases [1]. In this context, it is of great significance to study the impact of testicular aging on overall male health. The testes are core organs critical for maintaining male reproductive and endocrine functions. Their aging can lead to a decline in androgen levels and spermatogenic dysfunction, subsequently triggering a series of physiological and psychological issues that severely compromise the health and quality of life of middle-aged and elderly men [2]. Therefore, in-depth exploration of the mechanisms underlying testicular aging and the pursuit of effective intervention strategies have become an urgent research and clinical task.

Testicular aging encompasses not only age-related physiological decline but also premature and pathological aging caused by various pathogenic factors, reflecting distinct manifestations of the testes at different stages or levels of deterioration [3]. The factors contributing to testicular premature aging and pathological aging are complex and diverse, which can be divided into endogenous factors and exogenous factors. Endogenous factors originate within the organism, such as congenital genetic defects, dysregulation of intracellular molecular mechanisms, and the decline of physiological functions. These chiefly include genetic defects [4], Y‑chromosome microdeletions [5], imbalances in oxidative stress [6], DNA damage [7], and impairment of the blood–testis barrier [8], which together exacerbate cellular injury.Exogenous factors act on the testis through environmental exposure, lifestyle interventions, and acquired injuries, disrupting its structural and functional homeostasis and accelerating the processes of testicular degeneration and pathological aging. Environmental toxins such as di-(2-ethylhexyl) phosphate (DEHP) and decabromodiphenyl ether (DBDPE) [9, 10], as well as heavy metals like lead [11], can damage testicular cells. External factors, including trauma and radiation, may adversely affect the structural integrity and normal physiological functions of the testis. Unhealthy lifestyle choices, such as prolonged smoking [12], excessive alcohol consumption [13], and obesity [14], negatively impact testosterone secretion. Infections by pathogens such as the mumps virus can damage seminiferous tubules and LCs [8], while chemotherapeutic agents and hormonal medications inhibit the function of the hypothalamic-pituitary-gonadal (HPG) axis [15, 16], exacerbating the decline in testicular function and ultimately collaborating with endogenous factors to promote testicular aging.

Among the various changes associated with testicular aging, the functional decline of LCs is considered one of the central changes. LCs are the key cells in the testis responsible for the synthesis and secretion of androgens, and their dysfunction directly leads to androgen deficiency.A hallmark of testicular aging is androgen deficiency, which is not only a consequence but also a driver of testicular aging [17]. This bidirectional relationship underscores the critical role of maintaining testicular health in safeguarding overall male health. This paper reviews relevant research to summarize the effects of testicular aging on LCs and explores the application of stem cell technology in restoring Leydig cell(LC) function. It aims to propose new approaches and strategies for preventing and managing testicular aging in men, thereby improving the health and quality of life for middle-aged and elderly men. Given the growing understanding of aging mechanisms and the continuous advancements in science and technology, stem cell technology may offer high-quality, personalized therapeutic solutions for the aging population.

## Methods

This structured narrative review synthesized evidence identified through systematic searches of PubMed, Web of Science Core Collection, CNKI (China National Knowledge Infrastructure), and Google Scholar, with no start-date restriction and coverage through the most recently available records at the time of the review. Search strategies were tailored to each source and combined controlled vocabulary (e.g., MeSH/subject headings) with free-text terms related to testicular aging, Leydig cells, stem Leydig cells, mesenchymal stem cells, induced pluripotent stem cells, late-onset hypogonadism, testosterone deficiency, the testicular microenvironment, stem cell transplantation, and stem cell therapy. Eligible records comprised peer-reviewed human and preclinical studies, authoritative guidelines/consensus statements, and high-quality reviews that focused on mechanisms by which testicular aging affects Leydig cell function, applications of stem-cell approaches to restore Leydig cell function, and optimization strategies for stem-cell–based therapies; more recent publications and studies with higher methodological rigor (e.g., randomized controlled animal studies, standardized in vitro functional validation) were prioritized. We excluded articles unrelated to the core topic; non–peer-reviewed items (except authoritative guidelines/consensus); duplicates; records without full text (e.g., abstracts, conference papers, theses); and studies with unclear design, incomplete data, or lacking relevant mechanistic, efficacy, or safety outcomes. Titles/abstracts and then full texts were screened sequentially, with reference lists of key articles hand-searched to identify additional studies. Two reviewers independently conducted title/abstract screening and full-text assessment; disagreements were resolved by discussion or by consulting a third domain expert. Given substantial heterogeneity in study designs, models, and outcomes, no meta-analysis was performed and evidence was synthesized qualitatively. In total, 102 articles were included (see References), providing the basis for analyzing mechanisms by which testicular aging affects Leydig cells and the therapeutic potential of stem cell technologies.

### The broad impact of testicular aging on male health

Testicular aging affects various aspects of male health. As adult males age, testicular aging progresses, leading to a decline in the ability to synthesize and secrete testosterone. Testosterone deficiency can result in sexual dysfunction, reduced libido, erectile dysfunction, osteoporosis, muscle atrophy, increased fat accumulation, and metabolic disorders [18–20]. Testicular aging is also a key factor in the development of late-onset hypogonadism (LOH), which is characterized by low testosterone levels and associated clinical symptoms [21, 22]. The impact of testicular aging on male health extends beyond the physiological domain to include psychosocial aspects. Low testosterone levels are associated with psychological health issues such as depression, reduced motivation, and impaired concentration, which can further affect men’s quality of life and social functioning. Testosterone plays a critical role in maintaining bone density and muscle mass. In elderly men, testosterone deficiency may lead to osteoporosis and muscle weakness, increasing the risk of falls and fractures [23]. Moreover, testicular aging is a contributing factor to the onset of various chronic diseases. Studies have shown that low testosterone levels are linked to an increased risk of obesity, cardiovascular diseases, type 2 diabetes, and chronic kidney disease [24, 25]. Testosterone deficiency can disrupt lipid metabolism, reduce insulin sensitivity, and trigger inflammatory responses, thereby contributing to the development of these conditions. Understanding the effects of testicular aging on LCs is crucial for clarifying the changes in reproductive and endocrine functions during male aging. This knowledge also lays a solid foundation for the development of novel therapeutic strategies. Current treatment approaches include hormone replacement therapy, stem cell therapy, and other emerging technologies. These interventions aim to improve LC function and increase testosterone levels in the body, ultimately enhancing the quality of life and overall health of middle-aged and elderly men.

### The impact of testicular aging on serum testosterone levels and LOH

Testicular aging is a progressive decline in the structure and function of the testes as men age. This process leads to a reduced capacity of the testes to synthesize and secrete testosterone, and low testosterone levels can trigger late-onset hypogonadism (LOH). LOH is an age-related biochemical syndrome characterized by various clinical symptoms, including reduced libido, erectile dysfunction, depressed mood, fatigue, and decreased bone density, accompanied by significantly reduced serum testosterone (T) levels. According to multiple international guidelines (such as those from ISA, ISSM, EAU, EAA, and ASA), the diagnosis of LOH typically requires repeated measurements of serum total testosterone (TT) levels between 7:00 and 11:00 a.m. If serum TT consistently falls below approximately 7.0–11.0 nmol/L, or if further measurements of free testosterone (FT) based on sex hormone-binding globulin (SHBG) levels show FT below 225 pmol/L (approximately 6.5 pg/mL) and other potential causes of hypogonadism (e.g., thyroid disorders, depression) are excluded, a diagnosis of LOH can be confirmed in the context of the patient’s clinical symptoms. Currently, testosterone replacement therapy (TRT) is the primary treatment for LOH, often combined with lifestyle interventions and symptomatic management. However, TRT carries potential risks, including prostate enlargement, increased cardiovascular disease risk, and suppression of endogenous testosterone production, and its long-term safety remains uncertain [21]. The pathogenesis of LOH is closely associated with the decline in LC function, as LCs are the primary source of testosterone synthesis [22].

### Roles of Leydig cells in the male reproductive system and advances in research

**Fig. 1:**
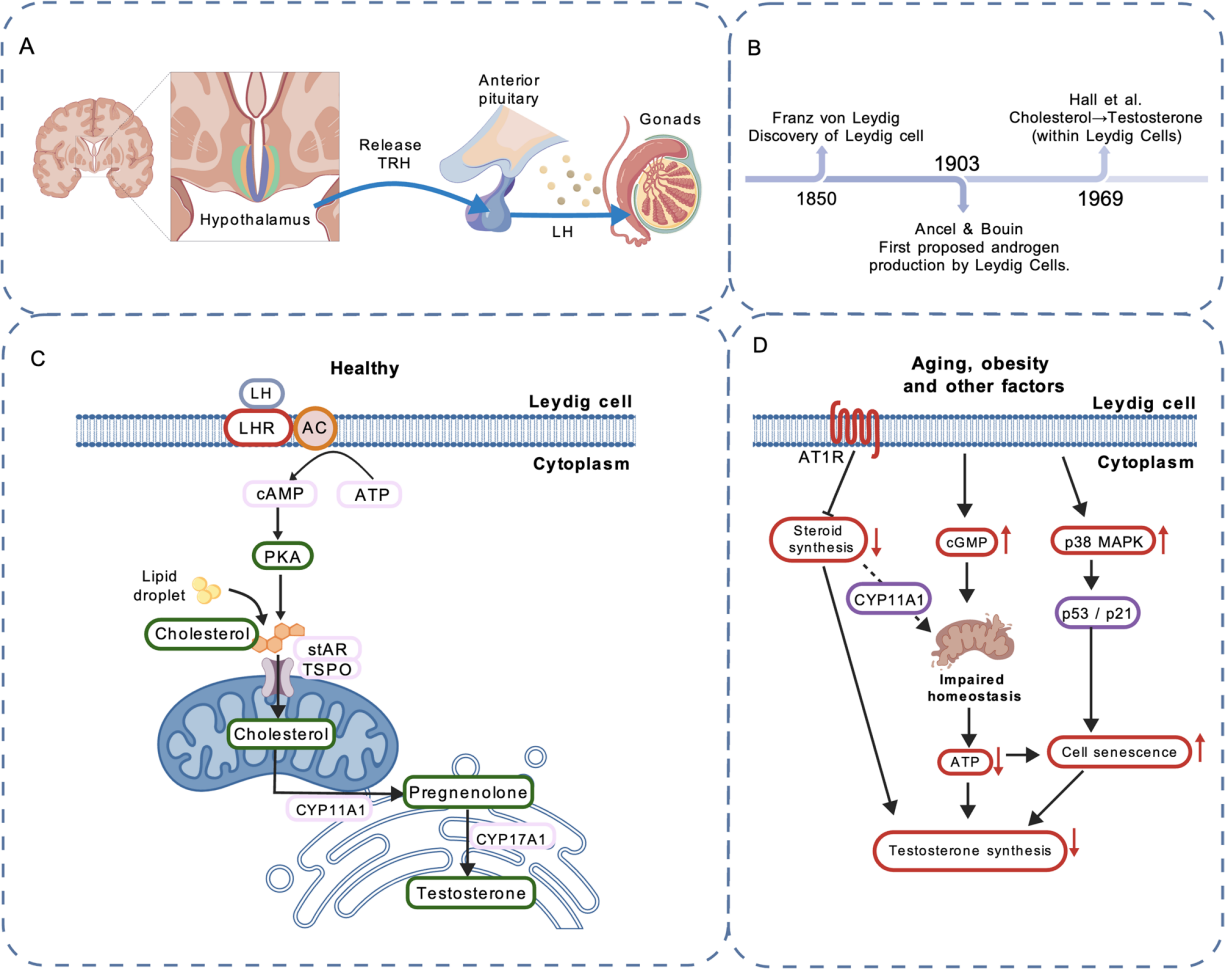
Advances in Leydig cell research. **A** Regulation of the hypothalamic–pituitary–gonadal (HPG) axis. **B** Historical milestones in LC research. **C** Testosterone biosynthetic pathway in LCs. **D** Current research on mechanisms related to LC senescence

Research on Leydig cells dates back to the 19th century (Fig. 1B). In 1850, the German histologist and anatomist Franz von Leydig first identified the testosterone-producing cells in the testis and named them Leydig cells [26]. In 1903, the French physiologists Ancel and Bouin first proposed that androgens are produced by LCs [27]. In 1969, Hall and colleagues demonstrated that the conversion of cholesterol to testosterone occurs within LCs [28].Testosterone production is regulated by the hypothalamic–pituitary–gonadal (HPG) axis. The hypothalamus secretes gonadotropin-releasing hormone (GnRH), which stimulates the pituitary to release luteinizing hormone (LH). LH binds to LH receptors on interstitial cells, triggering the production of cAMP, which in turn enhances the transport of cholesterol into mitochondria. Within mitochondria, cholesterol is converted to pregnenolone by the mitochondrial inner membrane enzyme CYP11A1. Pregnenolone is subsequently metabolized by enzymes located in mitochondria and the smooth endoplasmic reticulum to form testosterone (Figs. 1 A, C) [29].

Testosterone production occurs during both fetal and adult life, and the Leydig cell lineage comprises two distinct types over the course of development: fetal Leydig cells (FLCs) and adult Leydig cells (ALCs). FLCs secrete testosterone and thereby promote sex differentiation and the development of male reproductive organs during fetal life. After birth, FLCs in the testis decline and gradually disappear [30, 31]. Some studies suggest that a subset of FLCs may persist into adulthood [32, 33]; however, these cells are unlikely to contribute meaningfully to testosterone production in adults [34–37]. The origin and functions of FLCs and ALCs are distinct. The development of adult Leydig cells is independent of fetal Leydig cells; ALCs arise from stem cells [29, 38]. Work by Ren-Shan Ge and colleagues showed that interstitial cells in adult rats develop from stem cells present in the testis during early postnatal life (postnatal day 7) [39]. The principal function of ALCs is to synthesize and secrete testosterone, which plays essential roles in the development and maintenance of male secondary sexual characteristics, spermatogenesis, sexual function, fat distribution, bone health, muscle mass, and systemic metabolism [40, 41].However, LC function declines progressively with age in men, leading to reduced testosterone biosynthesis. This decline is both a hallmark and a consequence of testicular aging [17]. In adult men, serum testosterone levels decrease with advancing age after approximately 40 years, a phenomenon referred to as age-associated testosterone decline [21]. Reduced testosterone is associated with male infertility, sexual dysfunction, and age-related metabolic syndrome, collectively termed LOH [22].

Current research on LC aging and its mechanisms remains relatively limited. Under LH stimulation, LCs synthesize testosterone. This process depends on the steroidogenic acute regulatory protein (StAR) and a suite of steroidogenic enzymes, including CYP11A1, 3β-HSD, and CYP17A1 [42, 43]. Evidence indicates that with aging, LH levels in most men remain stable or increase slightly [44, 45]. The principal cause of impaired LC function is the downregulation of StAR and related steroidogenic enzymes [42].In a mouse model, Dandan Luo et al. [46] demonstrated that p38 mitogen-activated protein kinase (p38 MAPK) participates in aging- and obesity-induced LC senescence. Inhibition of p38 MAPK attenuated LC aging and mitigated the decline in testosterone levels. p38 MAPK, a member of the MAPK family, is involved in diverse cellular stress responses [47]. Oxidative stress and chronic inflammation associated with aging and obesity activate p38 MAPK [48]. The authors hypothesized that p38 MAPK may promote LC senescence via the p53/p21 signaling pathway; future studies are needed to elucidate the detailed mechanisms [46].Minye Zhao et al. [49] reported that expression of the angiotensin II type-1 receptor (AT1R) is markedly upregulated in testicular tissue samples from elderly men and rats. AT1R expression exerts an inhibitory effect on LC steroidogenesis and promotes senescence in smooth muscle and endothelial cells [50]. Angiotensin II (Ang II) mediates most actions of the renin–angiotensin system (RAS) primarily through activation of AT1R [51]. Their analyses indicated that inhibition of AT1R activity ameliorated LC aging and restored testicular steroidogenesis in aged rats, whereas forced AT1R expression in the testicular interstitium enhanced senescence-associated features in LCs. Thus, blockade of Ang II/AT1R signaling may help alleviate testicular aging [49].Studies by Srdjan J. Sokanovic et al. [52] in rat models showed that aging leads to aberrant elevation of cGMP signaling in LCs, disrupting mitochondrial homeostasis and resulting in reduced ATP production and steroidogenesis. Long-term PDE5 inhibition (sildenafil) decreased cGMP signaling in aged rat LCs and improved mitochondrial function and testosterone production [52]. These findings provide a new avenue for interventions targeting testicular aging (Fig. 1D).

### The impact of testicular microenvironment changes on Leydig cells during aging

As men age, the effects of testicular aging-induced changes in the microenvironment become increasingly evident. These changes in the testicular microenvironment are considered a critical factor contributing to Leydig cell dysfunction.

Curley et al. [53], using a Cisd2-deficient progeroid mouse model, demonstrated that aging of the testicular microenvironment impairs LC function. They observed testicular atrophy, reduced numbers of LCs and Sertoli cells (SCs), decreased circulating testosterone, and downregulation of steroidogenic gene expression—phenotypes resembling testicular functional decline in elderly men. However, LC- or SC-specific deletion of Cisd2 did not elicit overt pathology, indicating that LC dysfunction arises from global niche aging rather than cell-autonomous senescence, and that a youthful microenvironment exerts a protective effect on LCs. Dong S et al. [3] further reported that testicular aging is accompanied by multifaceted deterioration of the niche, including numerical and morphological abnormalities of SCs and LCs, organellar aging, disruption of the blood–testis barrier, and endocrine dysregulation; diminished SC function accelerates testicular aging via signaling pathways such as NFAT1/TXLNA and ERα/NRF2. Building on single-cell transcriptomics with machine-learning analyses, Cui L et al. [54] showed that somatic cells exhibit a stronger senescence response than germ cells and that aging proceeds in two stages: after age 30, aberrant extracellular matrix (ECM) signaling in testicular peritubular cells (TPCs) with basement membrane thickening marks the onset of aging; after age 50, LC steroidogenic capacity declines, with reduced expression of key genes (e.g., StAR, CYP11A1), accompanied by decreased testosterone, elevated gonadotropins, and macrophage dysfunction that drives chronic inflammation; moreover, obesity and advanced age synergistically exacerbate reproductive decline. Using single-cell sequencing coupled with functional validation, Nie X et al. [55] systematically delineated multidimensional degenerative changes in LCs and the testicular microenvironment during human testicular aging: older testes exhibit fewer LCs, aberrant upregulation of smooth muscle contraction–related genes, and impaired testosterone biosynthesis; SC numbers decrease with metabolic dysregulation; TPC numbers increase but their functions deteriorate; inflammatory responses are broadly heightened; intercellular communication is attenuated; and these changes are more pronounced in older individuals with high body mass index (BMI). Consistent with these findings, Xia K et al. [56] showed that aged LCs display upregulation of oxidative stress– and inflammation-related genes and downregulation of sterol biosynthesis and cholesterol metabolism genes, reflecting exacerbated oxidative stress and diminished steroidogenic capacity with aging. Collectively, these studies indicate that the global aging of the testicular microenvironment—rather than spontaneous decay of any single cell type—constitutes a multidimensional degenerative process and is a key driver of the age-associated decline in Leydig cell function.

These studies provide new perspectives for understanding the mechanisms of testicular aging and offer potential intervention strategies to delay LC functional decline by repairing the aging testicular microenvironment. Currently, stem cell technology has emerged as a promising strategy for restoring LC function during testicular aging [57]. Stem cells possess self-renewal and multipotent differentiation capabilities, allowing them to differentiate into Leydig-like cells, thereby overcoming the limitations of LC non-proliferation. Furthermore, stem cells can promote LC survival and functional recovery by secreting paracrine factors that improve the testicular microenvironment [58, 59].

### The potential of stem cell technology in restoring Leydig cell function

LCs are the primary site of testosterone synthesis. LC aging is one of the critical factors leading to testicular functional decline and reduced testosterone secretion [29, 40]. Theoretically, the ideal treatment for testosterone deficiency would involve the transplantation of functional LCs to restore testosterone secretion effectively. However, as LCs are terminally differentiated cells without proliferative capacity, it is challenging to obtain sufficient quantities of LCs for transplantation through in vitro expansion [29, 60]. Additionally, the direct acquisition of LCs faces technical and ethical challenges [61]. In recent years, research has focused on using stem cell technology to restore the testosterone-secreting function of LCs [62]. Stem cells possess strong self-renewal abilities and multipotent differentiation potential. Theoretically, transplanting stem cells into the testes could help improve the testicular microenvironment, promote LC regeneration, and restore LC function [63]. With the rapid development of gene engineering and biomaterials, several emerging strategies have been introduced to optimize therapeutic efficacy. These include preconditioning with physical and chemical factors, genetic modifications, and the integration of biomimetic materials, which have further enhanced the translational efficiency of stem cell technologies [64]. These advancements have not only explored new therapeutic approaches for testicular aging but also provided innovative strategies to improve LC function.

**Fig. 2:**
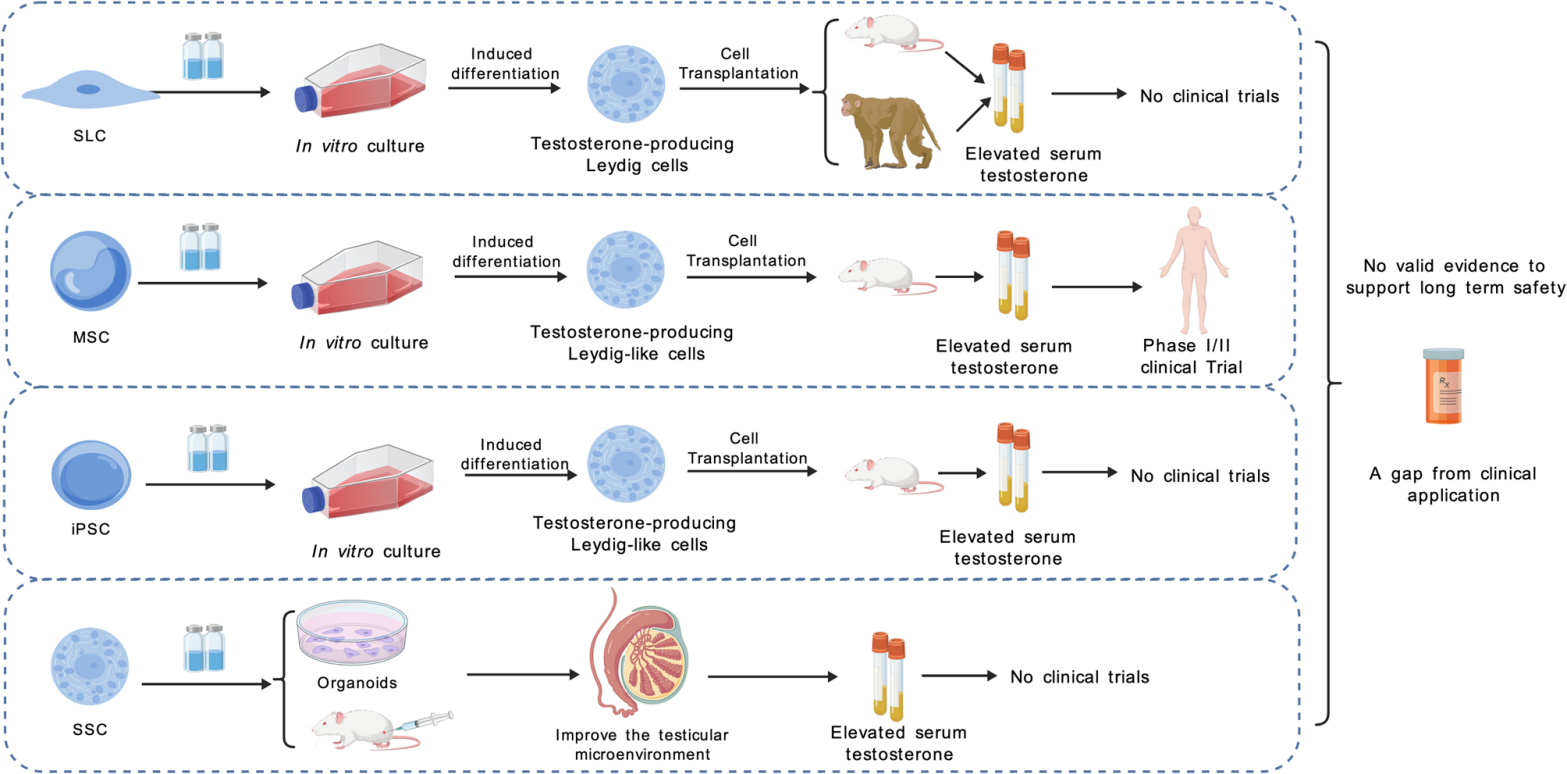
Types of stem cell therapies and current research progress

Researchers have successfully directed various types of stem cells to differentiate into Leydig-like cells, including stem leydig cells (SLCs), mesenchymal stem cells (MSCs), and induced pluripotent stem cells (iPSCs). SLCs are a type of adult stem cell found in the testicular interstitium with the potential to differentiate into mature LCs. As LCs are the primary source of testosterone in the body, the aging of SLCs, which leads to a decline in LC numbers, is a significant factor contributing to testicular aging [65, 66]. Studies have demonstrated that SLCs can differentiate into testosterone-producing Leydig-like cells in vitro, in rat models, and in nonhuman primate (NHP) models [67, 68]. However, challenges remain regarding the sourcing of SLCs, as well as the optimization of their in vitro expansion and differentiation efficiency [66]. Compared to SLCs, MSCs have advantages such as wide availability, ease of acquisition, and low immunogenicity, making them a current research hotspot [69]. MSCs can be induced in vitro to differentiate into Leydig-like cells and show potential to restore testosterone levels in rat models [70]. In 2021, Nguyen et al. reported that patients’ testosterone levels increased significantly following intravenous infusion of autologous adipose-derived MSCs, demonstrating that MSCs can be transplanted into humans and exert therapeutic effects. The study further suggested that MSCs may restore LC function through paracrine cytokine signaling and/or modulation of the immune system [71]. Somatic cells can be converted into iPSCs by introducing reprogramming factors using cellular reprogramming techniques. iPSCs are readily obtainable from diverse sources and exhibit high differentiation potential. Studies have shown that iPSCs can be differentiated in vitro into Leydig-like cells and, following transplantation into rat models, are capable of restoring serum testosterone levels [72]. However, the application of iPSCs currently faces challenges such as the risk of tumorigenesis and ethical concerns [58, 72]. In 2023, Jin C et al. found that spermatogonial stem cells (SSCs) can promote SSC maintenance, restore testicular injury, and improve the testicular microenvironment through paracrine actions in a mouse model [73]. SSCs are not only the initiating cells of spermatogenesis but also play a crucial role in regulating the testicular microenvironment [74, 75]. A summary of the foregoing is shown in Fig. 2. Stem cell technology holds significant potential for restoring LC function. However, challenges such as low differentiation efficiency, insufficient testosterone secretion capacity, and long-term safety concerns remain. In the future, the development of safe and efficient transplantation strategies may make stem cell technology a promising approach for addressing male testicular aging.

#### The role of stem Leydig cells in enhancing Leydig cell testosterone secretion

SLCs are tissue-specific adult stem cells located in the interstitial spaces surrounding seminiferous tubules and blood vessels in the testes. They possess self-renewal capabilities and the potential to differentiate into LCs [66, 76]. LCs are the primary cells responsible for synthesizing and secreting testosterone in the testes and are also an essential component of the spermatogenic microenvironment. Testosterone not only maintains male secondary sexual characteristics and sexual function but also plays a critical role in spermatogenesis [29]. With aging or the onset of diseases, the number and function of LCs gradually decline, leading to reduced testosterone secretion and impaired spermatogenic function [77]. Transplantation of SLCs, by virtue of their differentiation potential, provides a novel therapeutic strategy for restoring LC function and testosterone secretion. Following SLC transplantation, the proliferation and differentiation of endogenous Leydig stem cells may be activated [76]. In 2017, Zhang M et al. identified and isolated p75 neurotrophin receptor positive (p75+) SLCs from adult testes in in vitro culture, and confirmed their proliferative capacity and multilineage differentiation potential. Under induction conditions using culture medium containing LH, IGF‑1, and triiodothyronine (T3), p75 + SLCs differentiated in vitro into functional testosterone‑producing LCs. When transplanted into rat models, these cells restored serum testosterone levels and spermatogenesis in male rats [68]. In 2020, Xia K et al. first reported, in a non-human primate (NHP) model(cynomolgus monkey), that transplantation of SLCs not only significantly increased serum testosterone levels in injury-model animals, but also improved spermatogenesis and re-established testicular tissue homeostasis [67]. However, current research on SLC transplantation is largely focused on animal models, including rat and NHP models, and there are presently no studies on SLC transplantation in humans. For clinical translation, numerous challenges remain, such as the isolation and identification of SLCs, the efficiency of in vitro expansion, post‑transplant survival, and homing capacity, among others. Future research that delves deeper into the mechanisms of SLC differentiation and transplantation strategies may facilitate the clinical application of SLC transplantation for testicular aging-related diseases. This could provide innovative solutions for delaying testicular aging and restoring male reproductive health.

#### The role of mesenchymal stem cells in restoring Leydig cell testosterone secretion

MSCs, with their widespread availability, robust self-renewal capacity, and multipotent differentiation potential, have demonstrated significant research value in restoring LC function and testosterone secretion [78]. In 2020, Ji W et al. demonstrated that human umbilical cord–derived mesenchymal stem cells can be induced under in vitro culture conditions to differentiate into Leydig‑like cells capable of secreting testosterone [79]. In a phase I/IIa clinical study conducted in 2021, Nguyen et al. administered an intravenous infusion of autologous adipose‑derived MSCs to participants. The results showed that patients’ testosterone levels increased significantly after transplantation, remained elevated over the subsequent six months, and did not begin to decline until 12 months post‑treatment. Their study demonstrated that MSCs can be transplanted into humans and exert therapeutic effects [71]. Existing studies on MSCs also suggest that they may restore LC function through paracrine secretion of cytokines and modulation of the immune system. Compared to other cell types, MSCs have several advantages, including wide availability, strong expansion capacity, low immunogenicity, and adaptability to diverse tissue microenvironments [78]. However, challenges remain in improving the efficiency of MSC differentiation into Leydig-like cells and enhancing their functional capacity to secrete testosterone. Future research focusing on the mechanisms of MSC differentiation and the regulatory network of the testicular microenvironment may pave the way for MSCs to become a safe and effective cell-based therapeutic approach for restoring LC function and addressing testosterone deficiency.

#### The role of induced pluripotent stem cells in restoring Leydig cell testosterone secretion

iPSCs, with their remarkable multipotent differentiation potential, have shown significant research value in restoring LC testosterone secretion. iPSCs are derived from somatic cells through genetic reprogramming techniques and possess pluripotency similar to that of embryonic stem cells [80]. In 2019, Li L et al. demonstrated that iPSCs can be induced under in vitro culture conditions to differentiate into Leydig‑like cells capable of secreting testosterone [72]. Chen X et al. generated iPSC‑derived Leydig‑like cells in vitro and further showed in a rat model that transplantation of these iPSC‑differentiated Leydig‑like cells restored serum testosterone levels and increased testicular weight [58]. In 2021, Ishida T et al. reported and validated, in an in vitro study, a simple and stable protocol for directing iPSCs to Leydig‑like cells: doxycycline‑induced overexpression of NR5A1 combined with treatment with 8‑Br‑cAMP and forskolin efficiently differentiated iPSCs into testosterone‑secreting “Leydig‑like cells” [81]. At present, there are no studies on human transplantation of iPSC‑derived Leydig‑like cells. Compared to other stem cell types, iPSCs have more significant application potential due to their wide availability and the ability to be reprogrammed from a patient’s cells, thereby avoiding immune rejection. However, challenges remain in the differentiation of iPSCs into Leydig-like cells, including low differentiation efficiency, incomplete functionality, and the potential risk of tumorigenesis [57]. Researchers have employed strategies such as gene editing technologies (e.g., CRISPR/Cas9), optimization of differentiation media components, and the integration of biomaterials to enhance the efficiency and functionality of iPSC-derived Leydig-like cells [81, 82]. In the future, iPSCs hold promise as a novel therapeutic approach for treating testicular aging and related diseases.

#### The role of spermatogonial stem cells in enhancing Leydig cell testosterone secretion through paracrine effects

SSCs are undifferentiated germ cells located on the basement membrane of seminiferous tubules and serve as the initiating cells of spermatogenesis, responsible for sperm production and maintaining male fertility. SSCs possess the ability to self-renew and differentiate into mature sperm, and their dysfunction can lead to male infertility [83]. In recent years, studies conducted in vitro, in organ culture, and in animal models (primarily mouse and rat) have shown that SSCs not only play a critical role in spermatogenesis but also influence the testicular microenvironment via paracrine effects, thereby potentially exerting an indirect promotive effect on LC function [83, 84]. In 2023, Jin C et al. reported—based on mouse models and in vitro experiments—that Sertoli cells, Leydig cells, and peritubular myoid cells secrete factors such as GDNF and FGF2, which act on receptors on SSCs, including GFRA1/RET and FGFR, thereby regulating SSC self-renewal, proliferation, and differentiation. Somatic cells such as Sertoli cells, Leydig cells, macrophages, and peritubular myoid cells form a complex intercellular communication network with SSCs, jointly establishing the spermatogonial stem cell niche [73]. In in vitro culture, the use of advanced techniques such as nanofiber scaffolds can effectively expand SSCs while maintaining their functionality and genetic stability [85]. Through paracrine signaling, SSCs actively modulate the testicular microenvironment and indirectly promote the restoration of LC function. This finding expands our understanding of SSC functions.However, the application of SSCs faces significant challenges. SSCs are rare in vivo and difficult to isolate, and they tend to lose their stemness during in vitro culture. Furthermore, SSC transplantation is hindered by issues such as immune rejection and ethical concerns [86]. These limitations restrict the widespread application of SSCs in regenerative medicine and male infertility treatments.

#### Current issues and health risks associated with stem cell therapies

Different classes of stem cells possess salient characteristics such as high proliferative capacity, pluripotency, self-renewal, and broad differentiation potential, enabling them to treat diseases and injuries effectively; nevertheless, stem cell transplantation still entails substantial risks. Ethical and regulatory concerns, tumorigenicity, immune responses, cellular senescence, dose effects, and transplantation timing constitute the key risk domains that must be considered [87]. Accordingly, potential risks should be rigorously investigated before employing stem cell–based medicinal products in clinical phases and human trials.

Multiple issues and health risks remain: from ethical and regulatory perspectives, direct isolation of LCs presents technical hurdles and raises ethical and regulatory concerns; SLCs are not yet standardized with respect to sourcing, isolation/characterization, in vitro expansion, survival, and homing; iPSCs continue to raise ethical debate; SSCs are difficult to obtain, and transplantation raises ethical issues and risks of immune rejection [58, 60, 86]. Regarding tumor risk, this is closely linked to pluripotency, sustained proliferative capacity, and genetic/epigenetic stability; in particular, residual undifferentiated iPSCs can give rise to teratomas, and reprogramming-related factors may further increase tumorigenicity; iPSC-derived products therefore require stringent removal of undifferentiated cells and formal tumorigenicity testing, whereas other cell types (e.g., MSCs, SLCs, and SSCs) currently lack clinical evidence of tumorigenicity but warrant continued surveillance [87]. With respect to immunological risk, autologous cells are not inherently safe and allogeneic products are certainly not immune-privileged [88]; differences in differentiation state, repeated dosing, and administration timing can elicit or exacerbate immune rejection; although MSCs exhibit low immunogenicity and immunomodulatory properties, they are not fully immune-privileged, and allogeneic use and repeat administration may provoke immune responses [87]; SSC transplantation is subject to immune rejection; and human immunological evidence remains limited for SLCs, MSCs, and iPSC-derived Leydig-like cells. In terms of cellular senescence and functional stability, prolonged in vitro expansion can induce replicative senescence and functional decline, thereby attenuating therapeutic efficacy. As for dose and timing, no consensus exists on optimal dosing or standardized transplantation windows, and therapeutic outcomes are further influenced by dose uncertainty and the route of administration (intravenous [IV], intra-arterial [IA], intrathecal [IT], intramuscular [IM]). Reported serious adverse events include glial-like lesions with consequent paraplegia after allogeneic stem cell injections, intrarenal tumor formation following autologous hematopoietic stem cell injection, and severe visual deterioration up to blindness after intravitreal injection of autologous adipose-derived stem cells [88].

Overall, current research on stem cell technologies lacks standardized evidence generation and adverse-event reporting; interventions conducted outside formal clinical studies are not subject to mandatory reporting, likely leading to underestimation of harms. Randomized controlled trials are needed to assess the risk–benefit balance; otherwise, it is impossible to determine whether clinical benefit outweighs potential harm. Evidence regarding clinical-grade manufacturing and lot-to-lot quality consistency, long-term safety, and durable efficacy remains insufficient and should be strengthened through rigorous quality control and evidence-based studies within compliant regulatory frameworks to support standardized clinical translation.

### Optimization strategies for stem cell culture

The optimization strategies for stem cell culture can be broadly categorized into two main aspects: the application of traditional Chinese medicine (TCM) and modern biotechnology.

In the application of TCM, the use of herbal formulas, single herbs, and bioactive components to regulate signaling pathways and cytokine expression has been shown to promote stem cell proliferation, differentiation, and functional enhancement [89]. For instance, the kidney-tonifying herbal formula “Bushen Tianjing Fang” has been proven to activate the Wnt/β-catenin signaling pathway, thereby promoting the proliferation and osteogenic differentiation of mesenchymal stem cells (MSCs) [90]. Similarly, “Danggui Buxue Decoction” can regulate cytokine expression to promote MSC proliferation [91]. On the level of single herbs and their bioactive components, astragalus polysaccharides have been found to enhance MSC proliferation by increasing the expression of stem cell factors [92]. Salvia miltiorrhiza and epimedium have been demonstrated to promote MSC proliferation and differentiation [93]. With its multi-component and multi-target characteristics, TCM not only enhances the efficiency of stem cell culture but also improves its therapeutic efficacy. Future research should delve deeper into the specific regulatory mechanisms of TCM on various types of stem cells, facilitating its application in stem cell culture and regenerative medicine, thus providing a solid theoretical and practical foundation for advancements in this field.

In terms of modern biotechnology, preconditioning with physical and chemical factors such as hypoxic environments, fluid mechanical stimulation, and small molecule drugs—during in vitro culture can activate or regulate stem cell signaling pathways. This enhances their adaptability to in vivo stress environments and improves biological functions such as anti-apoptosis, homing, tissue repair, paracrine activity, and immunomodulation [94–96]. Gene engineering techniques, such as gene editing, can modify the expression of specific functional genes in stem cells, aiding in the elucidation of gene roles in disease or aging processes. These techniques also improve the therapeutic efficacy of stem cell transplantation by enhancing anti-inflammatory, anti-aging, and antioxidant functions [97, 98]. The application of biomimetic materials, which create three-dimensional culture systems that simulate in vivo microenvironments, provides biomechanical support for stem cells using biocompatible materials such as polylactic acid and gelatin. These systems promote cell adhesion, migration, and long-term engraftment while serving as diffusion media for paracrine factors, thereby enhancing stem cell environmental responsiveness and immunomodulatory functions [99–101]. Additionally, optimizing the composition of culture media by adding specific growth factors and cytokines—such as luteinizing hormone (LH), insulin-like growth factor-1 (IGF-1), and retinoic acid (RA)—and adjusting culture conditions such as temperature, pH, and oxygen concentration can significantly improve stem cell proliferation, self-renewal, and multipotent differentiation capabilities [58, 102].

The synergistic application of multiple optimization strategies has opened practical pathways for the large-scale in vitro expansion and functional enhancement of stem cells. This makes the large-scale production of autologous stem cells a feasible goal, further advancing the fields of regenerative medicine and cell therapy. Integrating the optimization strategies of TCM and modern biotechnology not only improves the efficiency and functional characteristics of stem cell cultivation but also lays a solid foundation for their application in regenerative medicine and clinical treatments. Future research should focus on exploring the synergistic effects of TCM and modern biotechnology, elucidating the molecular mechanisms of TCM in stem cell cultivation, optimizing the parameters for culturing various types of stem cells, and developing novel, highly efficient biomaterials. These efforts aim to achieve safer and more efficient stem cell culture and applications, ultimately contributing to breakthroughs in treating testicular aging and related male reproductive health issues.

## Conclusion

The decline in Leydig cell function is both a result of testicular aging and one of its key markers. Stem cell technology holds great potential in restoring Leydig cell function. Stem leydig cells, mesenchymal stem cells, induced pluripotent stem cells, and spermatogonial stem cells have demonstrated the ability to secrete testosterone or improve the testicular microenvironment under specific conditions. However, challenges remain in terms of differentiation efficiency, testosterone secretion capacity, and safety. By integrating traditional Chinese medicine with modern biotechnological approaches to optimize stem cell culture strategies; it is expected that breakthroughs can be achieved in the application of stem cell technology for treating testicular aging and related male reproductive health issues, providing new directions for clinical treatment.

## Data Availability

No datasets were generated or analysed during the current study.

## Abbreviations

LCs: Leydig Cells
LOH: Late-onset Hypogonadism
T: Testosterone
ISA: International Society of Andrology
ISSM: International Society for Sexual Medicine
EAU: European Association of Urology
EAA: European Anaesthesiology Association
ASA: American Society of Anesthesiologists
TT: Total Testosterone
FT: Free Testosterone
SHBG: Sex Hormone - Binding Globulin
TRT: Testosterone Replacement Therapy
LC: Leydig Cell
ROS: Reactive Oxygen Species
Cisd2: CDGSH iron-sulfur domain 2
SCs: Sertoli Cells,
BMI: Body mass index
SLCs: Stem Leydig Cells
MSCs: Mesenchymal Stem Cells
IPSCs: Induced Pluripotent Cells
SSCs: Spermatogonial Stem Cells
TCM: Traditional Chinese Medicine
LH: Luteinizing Hormone
IGF-1: Insulin-like Growth Factor-1
RA: Retinoic Acid
VEGF: Vascular Endothelial Growth Factor
FGF: Fibroblast Growth Factor
IGF: Insulin-like Growth Factor
DEHP: Di-(2-ethylhexyl) phosphate
DBDPE: Decabromodiphenyl ether
HPG: Hypothalamic-pituitary-gonadal
CNKI: China National Knowledge Infrastructure
GnRH: Gonadotropin-releasing hormone
FLCs: Fetal Leydig cells
ALCs: Adult Leydig cells
StAR: Steroidogenic acute regulatory protein
p38 MAPK: p38 mitogen-activated protein kinase
AT1R: Angiotensin II type-1 receptor
Ang II: Angiotensin II
ECM: Extracellular matrix
TPCs: Testicular peritubular cells
NHP: Nonhuman primate
p75+: p75 neurotrophin receptor positive
T3: Triiodothyronine
IV: Intravenous
IA: Intra-arterial
IT: Intrathecal
IM: Intramuscular
